# In Stallion Spermatozoa, Superoxide Dismutase (Cu–Zn) (SOD1) and the Aldo-Keto-Reductase Family 1 Member b (AKR1B1) Are the Proteins Most Significantly Reduced by Cryopreservation

**DOI:** 10.1021/acs.jproteome.0c00932

**Published:** 2021-03-03

**Authors:** Gemma Gaitskell-Phillips, Francisco E. Martín-Cano, José M. Ortiz-Rodríguez, Antonio Silva-Rodríguez, Maria C. Gil, Cristina Ortega-Ferrusola, Fernando J. Peña

**Affiliations:** † Laboratory of Equine Reproduction and Equine Spermatology, Veterinary Teaching Hospital, University of Extremadura, 10003 Cáceres, Spain; ‡ Facility of Innovation and Analysis in Animal Source Foodstuffs, University of Extremadura, 10003 Cáceres, Spain

**Keywords:** spermatozoa, cryopreservation, redox, proteomics, equids

## Abstract

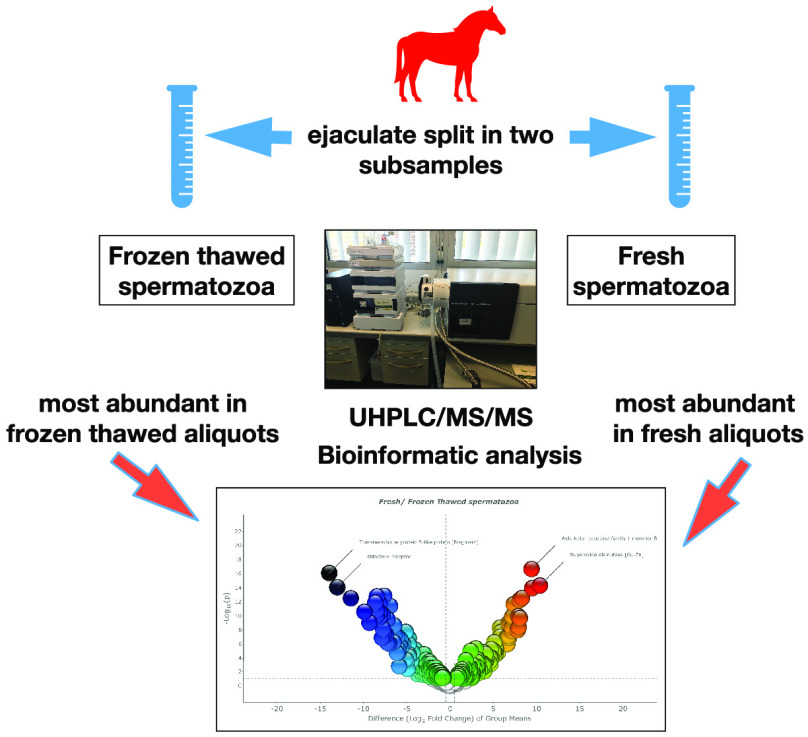

Although cryopreservation is widely used in animal breeding, the technique is still suboptimal. The population of spermatozoa surviving the procedure experiences changes attributed to alteration in their redox regulation. In order to expand our knowledge regarding this particular aspect, the proteome in fresh and frozen thawed aliquots of equine spermatozoa was studied to identify the proteins most severely affected by the procedure. If alteration of redox regulation is a major factor explaining cryodamage, proteins participating in redox regulation should be principally affected. Using a split sample design, 30 ejaculates from 10 different stallions were analyzed as fresh spermatozoa, and another aliquot from the same ejaculate was analyzed as a frozen thawed sample. The proteome was studied under both conditions using UHPLC-MS/MS and bioinformatic analysis conducted to identify discriminant variables between both conditions. Data are available through the ProteomeXchange Consortium with identifier PXD022236. The proteins most significantly reduced were *Aldo-keto reductase family 1 member B* (*p* = 2.2 × 10^–17^) and *Superoxide dismutase (Cu–Zn)* (*p* = 4.7 × 10^–14^). This is the first time that SOD1 has been identified as a discriminating variable using bioinformatic analysis, where it was one of the most highly significantly different proteins seen between fresh and frozen thawed semen. This finding strongly supports the theory that alteration in redox regulation and oxidative stress is a major factor involved in cryodamage and suggests that control of redox regulation should be a major target to improve current cryopreservation procedures.

## Introduction

In comparison with other domestic species, artificial insemination (AI) with frozen thawed spermatozoa is not as widely used in equine breeding. There are a number of factors that can explain this situation, among them past restrictions in the use of reproductive technologies by most stud books. As a result of these restrictions, in most breeds artificial insemination has been introduced in the past two decades. In comparison, artificial insemination has been widely used in bovines since the 1950s. As a consequence, research in equine reproductive biotechnologies is lagging a few decades behind. Perhaps the major constraint for the development of AI using equine frozen thawed semen is the stallion to stallion variability in cryotolerance.^1,2^ However, in spite of recent research,^3,4^ the reasons behind this variability remain largely unknown. Cryopreservation causes osmotic stress during freezing and especially during thawing, damaging all the sperm structures, including plasma and mitochondrial membranes.^4^ Osmotic effects account for sperm death due to acute necrosis; however, an important percentage of the surviving population experience accelerated aging leading to premature cell death. It is believed this process is due to redox imbalance and oxidative stress originating in the mitochondria. Advances in the identification of all the factors that control cryotolerance could be of significant importance for the equine industry. These are likely to serve as powerful tools for definition of specific targets which can be used to improve existing protocols used for cryopreservation. The introduction of the omics in spermatology is facilitating a rapid progression of knowledge of the biology of spermatozoa,^5−9^ and these technologies have recently been introduced as a tool for the study of the stallion spermatozoa.^10,11^ Proteomic technologies have been applied to investigate the changes induced by cryopreservation in different species,^11−18^ but to date, only one study^11^ has addressed the impact of cryopreservation in equine spermatozoa, revealing an important impact on proteins involved in both oxidative phosphorylation and redox regulation. This study focused on enrichment analysis of groups of proteins based on gene ontology terms and pathways analysis. The present study aimed to define the effects of cryopreservation on the equine sperm proteome.

## Materials and Methods

### Reagents and Media

Hoechst 33342 [(Ex: 350 nm, Em: 461 nm), (ref: H3570)] was purchased from Molecular Probes (Leiden, The Netherlands). Anti-4 hydroxynonenal (4-HNE) antibody [HNEJ-2] (ref: ab48506) and goat antimouse IgG (H&L) Alexa Fluor 647 [(Ex: 652 nm, Em: 668 nm) (ref: ab150115)] were purchased from Abcam (Cambridge, UK). All other chemicals were purchased from Sigma-Aldrich (Madrid, Spain), unless otherwise stated.

### Semen Collection and Processing

A sample of 10 stallions of different breeds was used to obtain semen. Animals were housed as required by specific institutional and European regulations for animal care (Law 6/2913 June 11th and European Directive 2010/63/EU). The study was approved by the University ethics committee. Collection of ejaculates was performed using a Missouri model artificial vagina, which was warmed and lubricated. An inline filter was used for removal of the gel fraction of the ejaculate. Upon collection semen was subsequently transferred straight to the laboratory where it was evaluated and processed. A split sample approach was used for experimental design, by dividing single ejaculates into two subsamples (fresh and frozen thawed experimental groups). Semen was processed in the laboratory using colloidal centrifugation^19,20^ to remove both dead spermatozoa and seminal plasma, and then either resuspended in Tyrodes media (20 mM HEPES, 5 mM Glucose, 96 mM NaCl, 15 mM NaHCO_3_, 1 mM Na-Pyruvate, 21.6 Na-Lactate, 2 mM CaCl_2_·2H_2_O, 3.1 mM KCl, 0.4 mM MgSO_4_·7H_2_O, 0.3 mM NaH_2_PO_4_·H_2_O, 0.3% BSA) 285 and 315 mOsm/kg at pH 7.4^21^ (fresh extended semen), or resuspended in cryopreservation media using standard procedures and protocols, as described in previous research by our group used for freezing (frozen thawed semen).^22^ Briefly, dilution of the aliquot was performed using the Cáceres freezing medium (University of Extremadura Cáceres, Spain), which is formulated from 2% egg yolk, 1% glycerol, and 4% dimethylformamide to 100 × 10^6^ spermatozoa/mL. Extended semen was loaded into 0.5 mL straws (IMV, L’Aigle, France), which were then sealed ultrasonically using an UltraSeal 21 (Minitube of America MOFA, Verona, Wisconsin, USA) machine, after which they were immediately transferred to an IceCube 14S (SY-LAB Neupurkersdorf, Austria) programmable freezer. The freezing curve used followed the subsequent steps. Straws were first kept at 20 °C for 15 min, after which they were then cooled slowly from 20 to 5 °C at a cooling rate of 0.1 °C/min. The freezing rate was then increased to −40 °C/min to take the temperature from 5 °C to −140 °C. After completion of the freezing curve straws were then plunged into liquid nitrogen and stored until further analysis. Thawing of frozen straws was performed using a water bath at 37 °C for at least 30 s.

### Experimental Design

Ejaculates were collected from 10 different stallions on 3 different occasions and collection and processing was as follows. Ejaculates were divided into two, and half of each ejaculate was frozen using standard protocols previously described in our laboratory (frozen thawed), while the other half was processed as for fresh spermatozoa (fresh).

### Sperm Preparation

Both fresh and frozen thawed (FT) samples of spermatozoa were washed three times using PBS (600*g* × 10 min). After this, samples were subsequently pelleted and kept frozen at −80 °C until further analysis. Phase contrast microscopy was used to ensure purity of the samples.

### Protein Solubilization

Lysis buffer (C7:C7Bz0 [3-(4-heptyl) phenyl-(3-hydroxypropyl) dimethylammoniopropanesulfonate], 7 M urea, 2 M thiourea, and 40 mM Tris (pH 10.4)) was used to solubilize isolated spermatozoa (200 × 10^6^ spermatozoa). Twenty microliters of lysis buffer were added for every 10 × 10^6^ spermatozoa, which were then vortexed and incubated while under constant rotation at −4 °C for 1 h.

### Protein Quantification

The manufacturer’s instructions (https://www.gelifesciences.co.jp/tech_support/manual/pdf/80648622.pdf) were followed while using the 2-D Quant Kit (GE Healthcare, Sevilla Spain) for protein quantification. All samples were then normalized in order to obtain a final protein concentration of 100 μg per sample.

### In-Solution Trypsin Digestion

100 μL of 25 mM ammonium bicarbonate buffer pH 8.5 (100 μg of protein in 300 μL of solution) was mixed with 200 μL of solution obtained from the previous protein solubilization stage. Proteins were reduced in this solution by the addition of 30 μL of 10 mM DTT after which they were incubated at 56 °C for 20 min. Alkylation of proteins was then performed by adding 30 μL of 20 mM IAA with subsequent incubation for 30 min at room temperature (r.t.) in the dark. Lastly, 1 μL of Trypsin Proteomics grade (Sigma) (Trypsin solution: 1 μg/μL in 1 mM HCl) was added for digestion of proteins for at least 3 h to overnight at 37 °C. Ten μL of 0.1% formic acid was used to stop the reaction and samples were filtered through 0.2 μm (hydrophilic PTFE) into 2 mL dark glass vials. To complete the process, samples were dehydrated using a nitrogen current with the vial placed in a heating block at 35 °C. Dry samples were then resuspended in 20 μL of buffer A, consisting of water/acetonitrile/formic acid (94.9:5:0.1)

### UHPLC-MS/MS Analysis

A UHPLC-MS system consisting of an Agilent 1290 Infinity II Series UHPLC (Agilent Technologies, Santa Clara, CA, USA) fitted with an automated multisampler module and a high speed binary pump, coupled to an Agilent 6550 Q-TOF Mass Spectrometer (Agilent Technologies, Santa Clara, CA, USA) using an Agilent Jet Stream Dual electrospray (AJS-Dual ESI) interface was used to separate and analyze the samples. MassHunter Workstation Data Acquisition software (Agilent Technologies, Rev. B.06.01) was used to control the HPLC and Q-TOF. Samples were injected onto an Agilent AdvanceBio Peptide Mapping HPLC column (2.7 μm, 150 × 2.1 mm, Agilent technologies), appropriate for peptide separation and analysis, at a flow rate of 0.4 mL/min and thermostatted at 55 °C. Operation of the mass spectrometer was in positive mode and a gradient program was used starting with 2% of B (buffer B: water/acetonitrile/formic acid, 10:89.9:0.1) remaining in isocratic mode for 5 min, increasing linearly up to 45% B over a period of 40 min, after which it was further increased up to 95% B over a time frame of 15 min and then kept constant for 5 min. The initial condition for column conditioning was used for 5 min of post-time after this 65 min run prior to the next run. Nebulizer gas pressure was set to 35 psi, and drying gas flow was set to 10 L/min at a temperature of 250 °C, with sheath gas flow set to 12 L/min at 300 °C. Capillary spray, fragmentor and octopole RF Vpp voltages were 3500 V, 340 and 750 V respectively. Profile data were acquired for both MS and MS/MS scans in Extended dynamic range mode^23,24^ was used for acquisition of profile data for both MS and MS/MS scans to eradicate potential contaminants. MS and MS/MS scan rates were 8 spectra/sec and 3 spectra/sec respectively with a mass range of 50–1700 *m*/*z*. Precursor selection by abundance and a maximum of 20 precursors selected per cycle were used in auto MS/MS mode. A ramped collision energy was used with a slope of 3.6 and an offset of −4.8. The same ion was rejected after two consecutive scans.

### Data Processing

Spectrum Mill MS Proteomics Workbench (Rev B.04.01, Agilent Technologies, Santa Clara, CA, USA) was used for processing and analysis of data. In summary, default conditions were used for extraction of raw data as follows: nonfixed or variable modifications were selected; [MH]^+^ 50–10 000 *m*/*z*; maximum precursor charge +5; retention time and *m*/*z* tolerance ±60 s; minimum signal-to-noise MS (S/N) 25; finding ^12^C signals. The following criteria were used for the MS/MS search against the appropriate and updated protein database (in this case: Uniprot/Horse): selection of nonfixed modifications with the following selected as variable modifications: carbamidomethylated cysteines and tryptic digestion with 5 maximum missed cleavages; ESI-Q-TOF instrument, minimum matched peak intensity 50%, maximum ambiguous precursor charge +5, monoisotopic masses, peptide precursor mass tolerance 20 ppm, product ion mass tolerance 50 ppm, and calculation of reversed database scores. Validation of peptide and protein data was performed using the autovalidation algorithm. This is completely automated and used to validate the highest-scoring results; those which do not require manual review are considered high-quality results. The autovalidation strategy used was autothreshold, in which the peptide score is automatically optimized for a target % FDR (1.2%). Protein polishing validation was then performed in order to increase the sequence coverage of validated results with the restriction of a new maximum target protein FDR (0%).

### Bioinformatics

#### Variance Filtering and PCA

Data were normalized and log transformed. Variables with low overall variance were filtered out to reduce the impact of noise, and the remaining variables were then centered and scaled to zero mean and unit variance, after which Principal Component Analysis (PCA) was used for visualization of the data set in a three-dimensional space. The optimal filter threshold was established using the projection score.^25,26^ Qlucore Omics Explorer version 3.6 Lund Sweden (https://qlucore.com) bioinformatics software was used for analysis. Hierarchical clustering and heat maps were used to display protein expression patterns^27^ and t-SNE (t-statistic Stochastic Neighbor Embedding) maps of standardized samples were used to identify relations between samples.^28,29^

#### Identification of Discriminating Variables

Qlucore Omics Explorer (https://qlucore.com) was used for identification of discriminating variables that are most highly significantly different between the two subgroups, fresh and frozen thawed spermatozoa. Identification of significantly different variables between the subgroups of fresh and frozen thawed spermatozoa from every single ejaculate was undertaken by fitting a linear model for each variable with each condition as a predictor and including the stallion nuisance covariate. The Benjamini–Hochberg method^30,31^ was used for multiple testing with adjusted *p*-values, and variables with adjusted *p*-values below 0.1 were considered significant.

#### Enrichment Analysis

Enrichment analysis was performed using the g:Profiler web server (https://biit.cs.ut.ee/gprofiler/gost)^32^ of the most abundant proteins in fresh or frozen thawed ejaculates. Electronically inferred annotations were excluded from enrichment analysis and focus on annotations with stronger evidence when higher confidence is wanted for the enrichment results. Only annotated genes were used and the g:SCS threshold was set at *P* < 0.01.

### Spermatozoa Motility

A Computer Assisted Sperm Analysis (CASA) system (ISAS Proiser, Valencia, Spain) was used to assess sperm motility and kinematic parameters as previously described.^33−35^ In brief, a Leja chamber with a depth of 20 μm (Leja, Amsterdam, The Netherlands) was placed on a warmed stage, at 38 °C and loaded with semen. Evaluation of 60 consecutive digitalized images obtained using a 10× negative phase-contrast objective (Olympus CX41) was used for analysis. A minimum of three different fields were captured, ensuring that at least 500 spermatozoa were analyzed per sample. Spermatozoa with a VAP (average velocity) < 15 μm/s were considered immotile, while spermatozoa with VAP > 15 μm/s were considered motile. Spermatozoa deviating <45% from a straight line were classified as linearly motile.

#### Measurement of Lipid Peroxidation

Two μL/mL of a stock solution of 0.1 mg/mL of anti 4-HNE primary antibody was used to stain spermatozoa (5 × 10^6^/mL) in 1 mL of PBS and these were then incubated at r.t. in the dark for 30 min. PBS was then used to wash cells and these were stained with 2 μL/ml of secondary Anti mouse Alexa Fluor 647 antibody (Excitation 650 nm, Emission 665 nm) and 0.2 μM Hoechst 33342 (Excitation 350 nm, Emission 461 nm) and incubated for a further 30 min in the dark at r.t.. Cells were then washed in PBS and samples were run immediately through the flow cytometer (Cytoflex LX flow cytometer Beckman Coulter, Brea, CA, USA), equipped with ultraviolet, violet, blue, yellow, red, and infrared lasers. Daily calibration of the instrument was performed using specific calibration beads provided by the manufacturer. A compensation overlap was performed before each experiment. Files were exported as FCS files and analyzed using FlowjoV 10.7.1 Software for Mac (Ashland, OR, USA). Controls consisted of unstained, single stained, secondary only antibody staining, and fluorescence minus one (FMO) controls, to ensure gates and compensations were properly set. Positive controls for 4-HNE were samples supplemented with 800 μM SO_4_Fe and 1.7 M of H_2_O_2_ (Sigma) to induce the Fenton reaction. Debris were gated out based on DNA content of the events after H33342 staining.^36,37^

### Statistical Analysis

GraphPad Prism version 7.00 for Mac, La Jolla, CA, USA, www.graphpad.com was used for statistical analysis. Fresh and frozen thawed samples were compared using a two tailed Mann–Whitney test. Differences were considered significant when *p* < 0.05, and results are displayed as means ± SEM.

## Results

### Cryopreservation Impacts the Stallion Sperm Proteome

Identification and quantification of a total of 910 different proteins was performed. The mass spectrometry proteomics data have been deposited in the ProteomeXchange Consortium via the PRIDE^38^ partner repository with data set identifier PXD022236.

**Figure 1:**
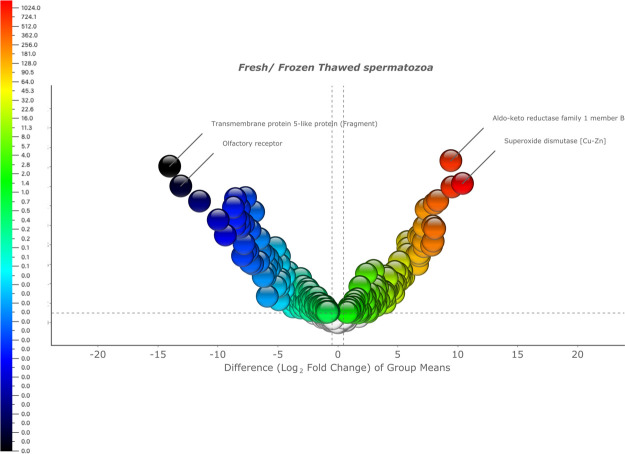
Volcano plot showing changes in the stallion sperm proteome as a consequence of cryopreservation. Proteins which were more abundant in fresh samples (and less abundant in frozen thawed samples) are presented on the right-hand side of the volcano plot, proteins most abundant in frozen and thawed samples (and less abundant in fresh samples) are presented on the left-hand side of the volcano plot. The two most significantly different proteins between the two conditions (higher fold change and higher *p* and *q* values) are depicted. The difference of protein content (log_2_ fold change) is plotted against the significance of the difference −log_10_(*p*) between the two conditions (fresh and frozen thawed spermatozoa) (3 independent ejaculates from 10 different stallions in addition to two technical replicates *n* = 60 samples were used to derive results from).

**Figure 2:**
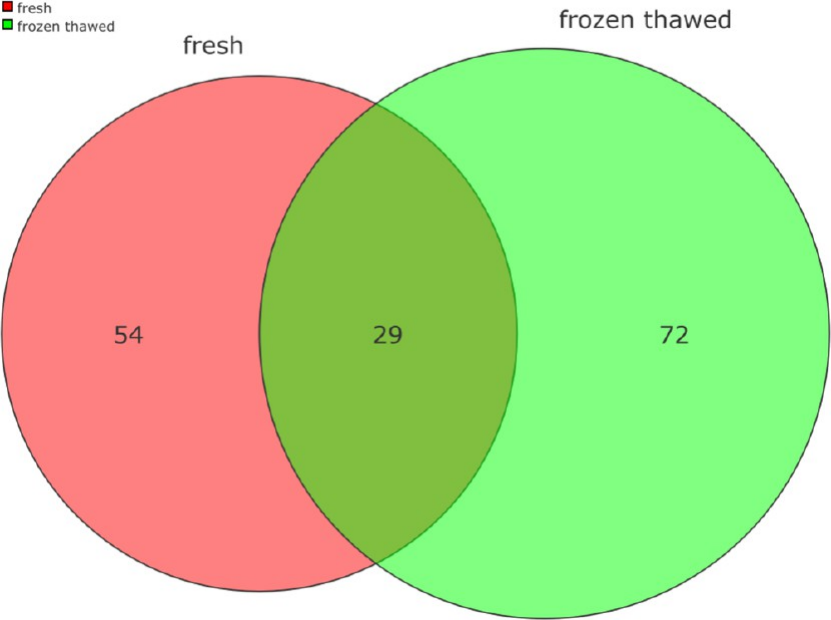
Venn diagram showing the overall impact of cryopreservation on the proteome of stallion spermatozoa. The number of proteins present in significantly different amounts (either increased or decreased) in both conditions are presented in the diagram. On the left, the number of proteins present in significantly different amounts in fresh semen, on the right the number of proteins present in significantly different amounts in frozen thawed samples.

**Figure 3:**
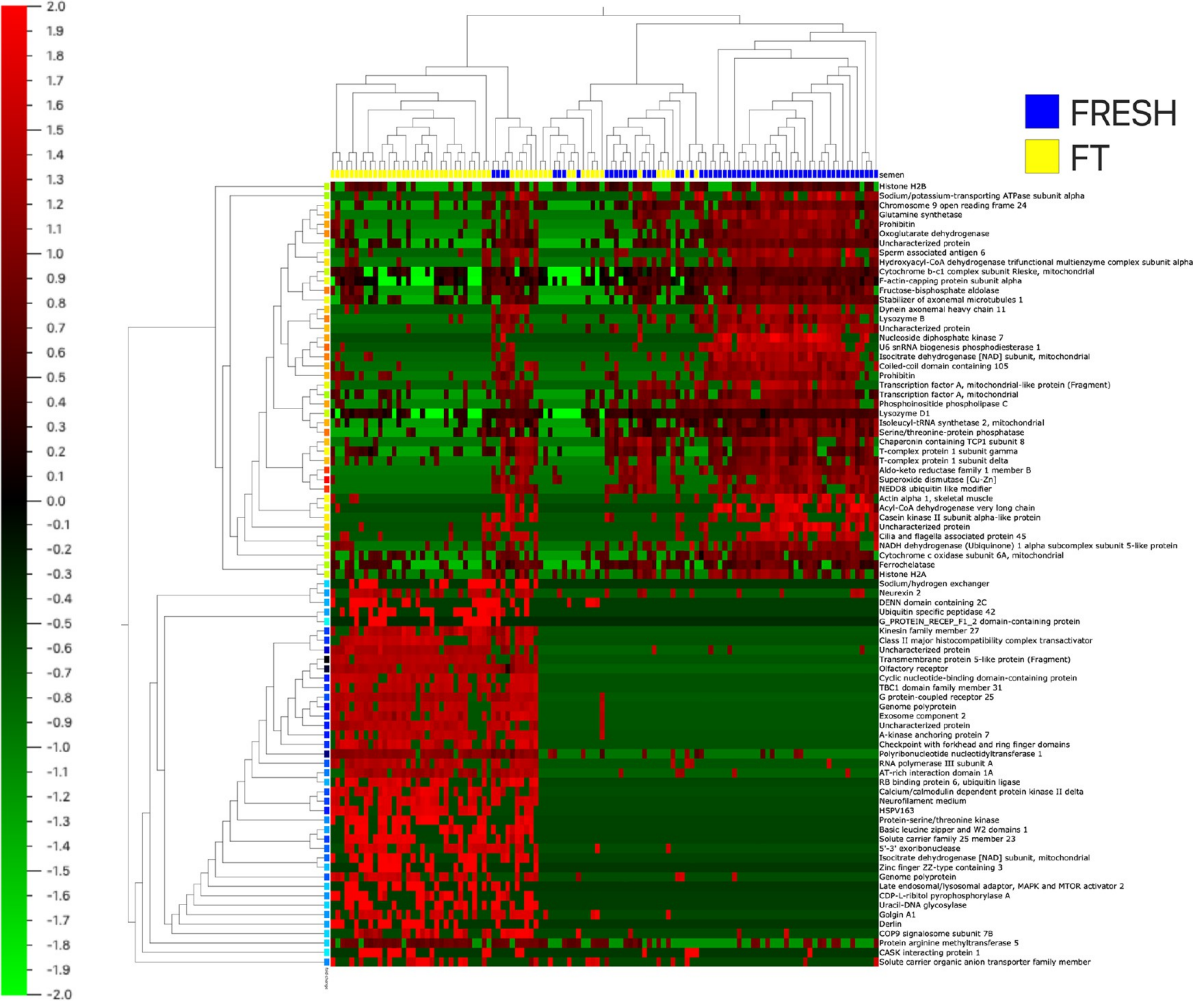
Heat map showing the impact of cryopreservation on the proteome of stallion spermatozoa. Proteins are classified following hierarchical clustering. Blue marks correspond with fresh samples, yellow marks correspond with frozen thawed samples. The heat map code is present with red areas representing greater amounts of protein and green areas lesser amount of protein. Proteins were normalized and filtered by a fold change >3 with *p* = 0.04 and *q* = 0.1.

**Figure 4:**
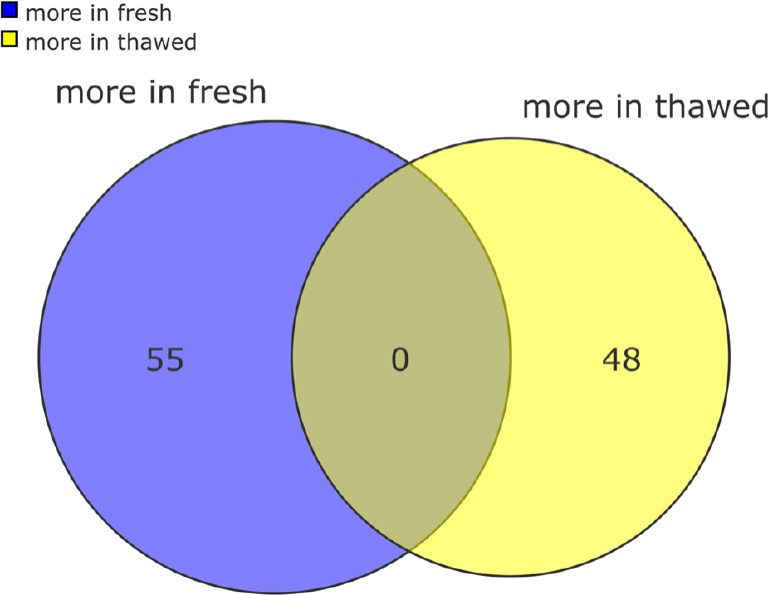
Venn diagram showing changes in the stallion sperm proteome as a consequence of cryopreservation. The number of proteins most abundant in each of the conditions are shown. On the left 55 proteins were more abundant in fresh spermatozoa, on the right 48 proteins were more abundant in frozen and thawed spermatozoa.

**Figure 5:**
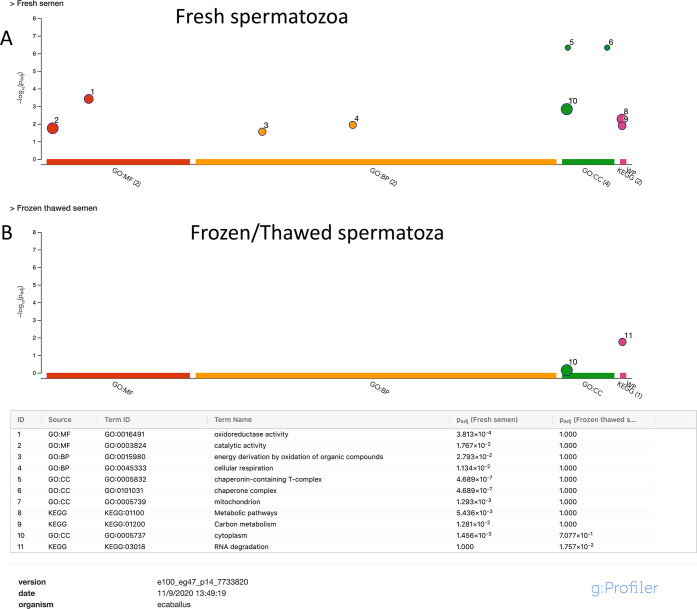
g:GOST multiquery Manhattan plot showing enrichment analysis of proteins present in higher amounts in fresh (A) and frozen thawed samples (B). The sperm proteome under each condition was queried against the equine proteome database. Gene Ontology (GO) terms for molecular function (MF) are in red, those for biological process (BP) in orange, and those for cellular component (CC) in green. KEGG pathways are depicted in red. The *p* values are depicted on the *y* axis and in more detail in the results table below the image.

Cryopreservation caused an important impact on the stallion sperm proteome (Figure 1). A Volcano plot revealed that numerous proteins experienced either increases or decreases in levels as a result of cryopreservation. Venn diagrams were used to identify the number of proteins varying between both conditions (Figure 2). Following this a two group (fresh vs frozen thawed) analysis was performed and changes identified in the proteome with a fold change >2 with *p* = 0.04 and *q* = 0.1, obtaining 102 proteins affected by the procedure. In the heat map in Figure 3, changes in the stallion proteome as a consequence of cryopreservation are represented. Another Venn diagram was then built to identify proteins changing during the procedure and proteins which were abundant under both fresh and frozen thawed conditions (Figure 4). In order to better identify changes caused by cryopreservation, enrichment analysis in both groups using g profiler was performed. In fresh semen, the proteins identified were enriched with the GO terms and KEGG pathways related to *oxidoreductase activity*, *cellular respiration*, *mitochondria*, *chaperone complex*, and *metabolic pathways* (Figure 5). In proteins which were most abundant in frozen thawed samples, the Kyoto encyclopedia of genes and genomes (KEGG) pathway RNA degradation (KEGG:03018) was significantly enriched (Figure 5).

**Figure 6:**
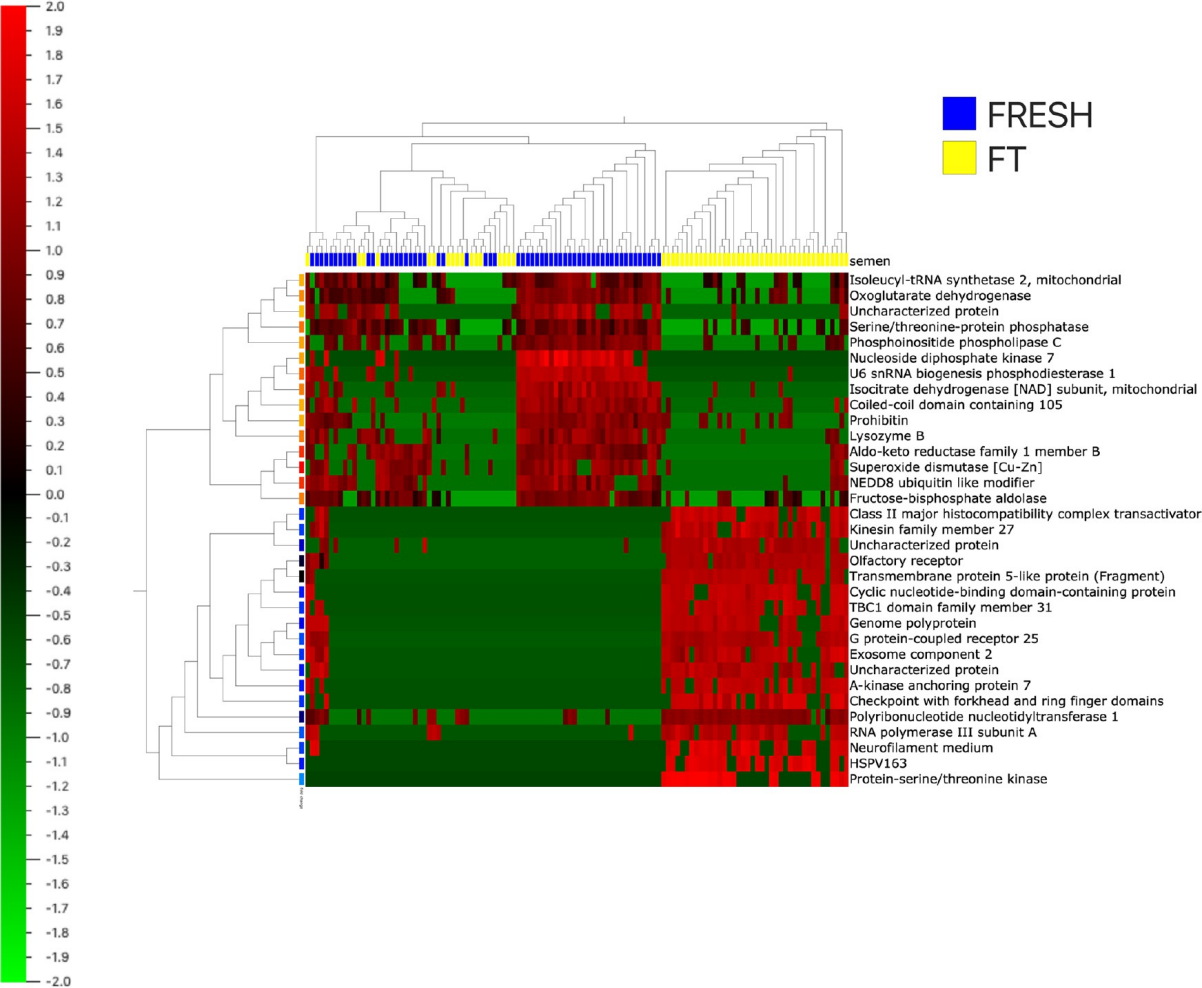
Heat map showing discriminant variables between fresh and frozen thawed spermatozoa. Proteins are classified following hierarchical clustering. Blue marks correspond with fresh samples, yellow marks correspond with frozen thawed samples. The heat map code is present with red areas representing greater amounts of protein and green areas lesser amounts of protein. Proteins were normalized and filtered by a fold change >3.75 with *p* = 1.15 × 10^–18^ and *q* = 3.6 × 10^–8^.

**Figure 7:**
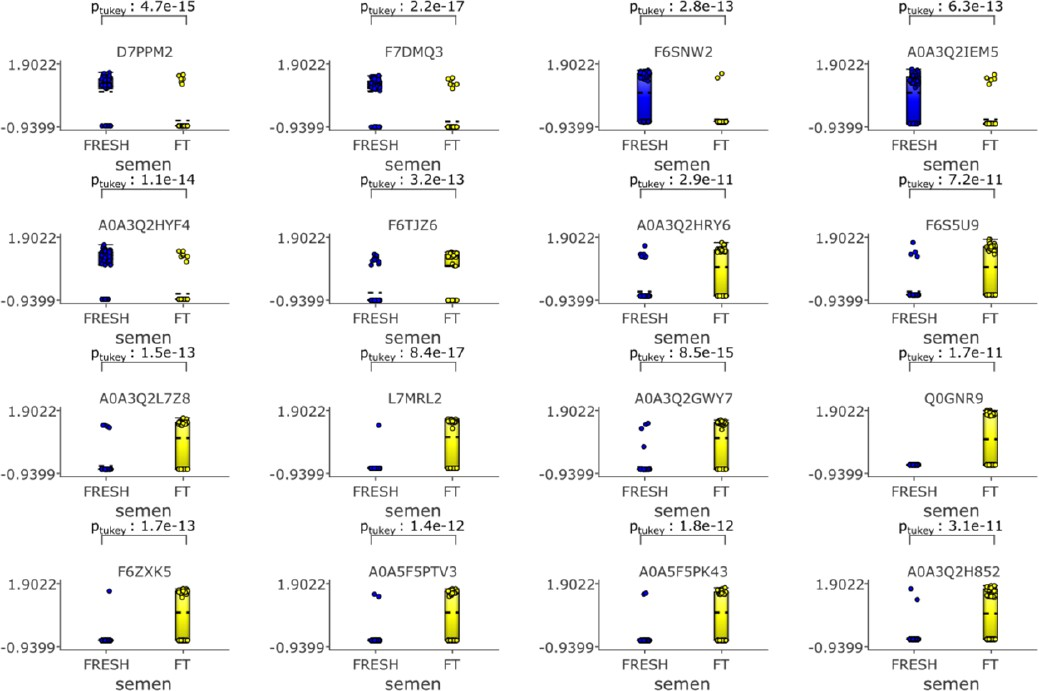
Differences in the amount of specific representative proteins under two conditions, fresh and frozen thawed spermatozoa. Qlucore Omics Explorer (Lund, Sweden https://qlucore.com) was used to compare differences in the relative amounts of proteins based on spectral counts between fresh and frozen thawed spermatozoa. Proteins in fresh spermatozoa are represented by blue circles, proteins in frozen thawed spermatozoa are represented by yellow circles. Proteins were normalized and filtered by a fold change >4 with *p* = 1.15 × 10^–18^ and *q* = 3.6 × 10^–8^.

**Figure 8:**
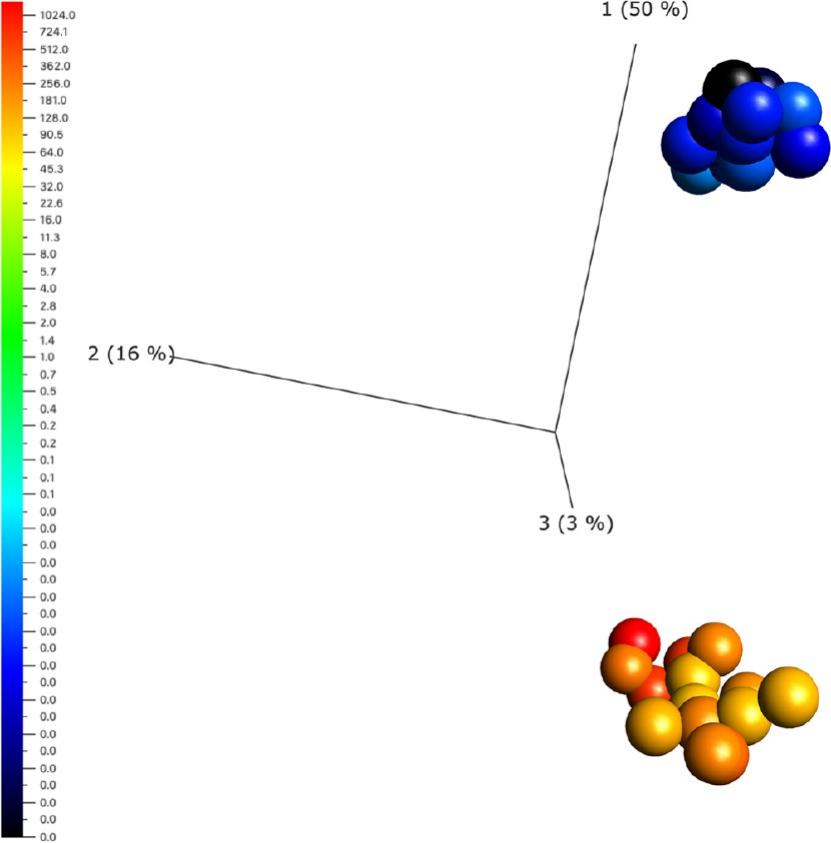
3D principal component analysis (PCA) of the variables affected by cryopreservation. Two groups are clearly seen representing proteins in which amounts increase after cryopreservation (yellow-orange) and those in which amounts are reduced as a consequence of cryopreservation (blue-black). Variables were prefiltered by standard deviation (S/S_max_) 0.6 and were then normalized and filtered by a fold change >4 with *p* = 1.15 × 10^–18^ and *q* = 3.6 × 10^–8^.

**Figure 9:**
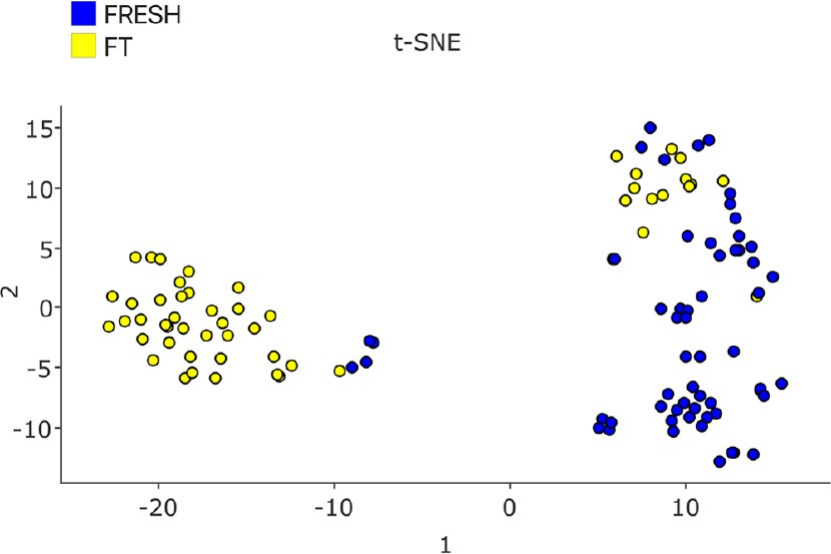
t-Distributed stochastic neighbor embedding (t-SNE), machine learning algorithm for visualization based on Stochastic Neighbor Embedding. The t-SNE map shows the distribution of the samples based on their proteome. After t-SNE clustering most of the fresh samples and the frozen thawed samples are classified in the same cluster. Fresh spermatozoa are shown in blue and frozen thawed in yellow.

In order to reduce the number of proteins retrieved, the threshold for the fold change was further increased and the *q* value reduced, leading to identification of 33 proteins in which amounts in the stallion spermatozoa were significantly affected by the cryopreservation procedure with a fold change >4 with *p* = 1.15 × 10^–18^ and *q* = 3.6 × 10^–8^. These included proteins whose levels either increased or decreased as consequence of cryopreservation (Figure 6 and 7). A separate analysis for proteins whose level increased or decreased after cryopreservation was also performed. A 3D principal component analysis (PCA) was then conducted of the variables most significantly affected by the procedure revealing two highly differentiated groups of proteins (Figure 8). A t-SNE analysis was also applied to the individual ejaculates, revealing two distinct populations, with the majority corresponding to either fresh or frozen thawed aliquots (Figure 9).

### Cryopreservation Reduces the Amount of Nine Sperm Proteins

**Figure 10:**
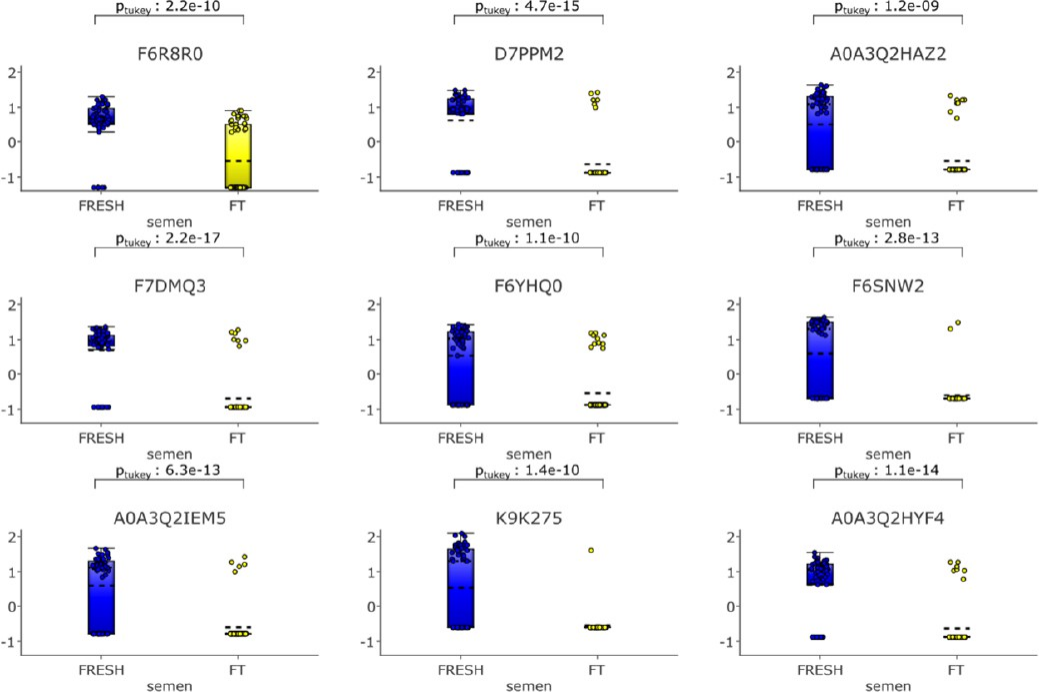
Proteins which are more abundant in fresh samples filtered by a fold change >4 with *p* = 6.4 × 10^–10^ and *q* = 7.51 × 10^–9^. Qlucore Omics Explorer (Lund, Sweden https://qlucore.com) was used to compare differences in the relative amounts of proteins based on spectral counts between fresh and frozen thawed spermatozoa. Proteins in fresh spermatozoa are represented by blue circles, proteins in frozen thawed spermatozoa are represented by yellow.

The amount of nine sperm proteins was significantly reduced as a consequence of cryopreservation (fold change >4, *p* = 6.40 × 10^–10^ and *q* = 7.51 × 10^–9^). These proteins were *superoxide dismutase (Cu–Zn)*, *serine/threonine-protein phosphatase*, *an uncharacterized protein (A0A3Q2HAZ2)* belonging to the actin family, *aldo-keto reductase family 1 member B*, *lysozyme B*, *U6 snRNA biogenesis phosphodiesterase 1*, *isocitrate dehydrogenase (NAD) subunit*, *mitochondrial*, *nucleoside diphosphate kinase 7*, and *NEDD8 ubiquitin like modifier*. The proteins most significantly reduced were *aldo-keto reductase family 1 member B* (*p* = 2.2 × 10^–17^) and *superoxide dismutase (Cu–Zn)* (*p* = 4.7 × 10^–14^) (Figure 10).

### Cryopreservation Increases the Amount of 16 Proteins

**Figure 11:**
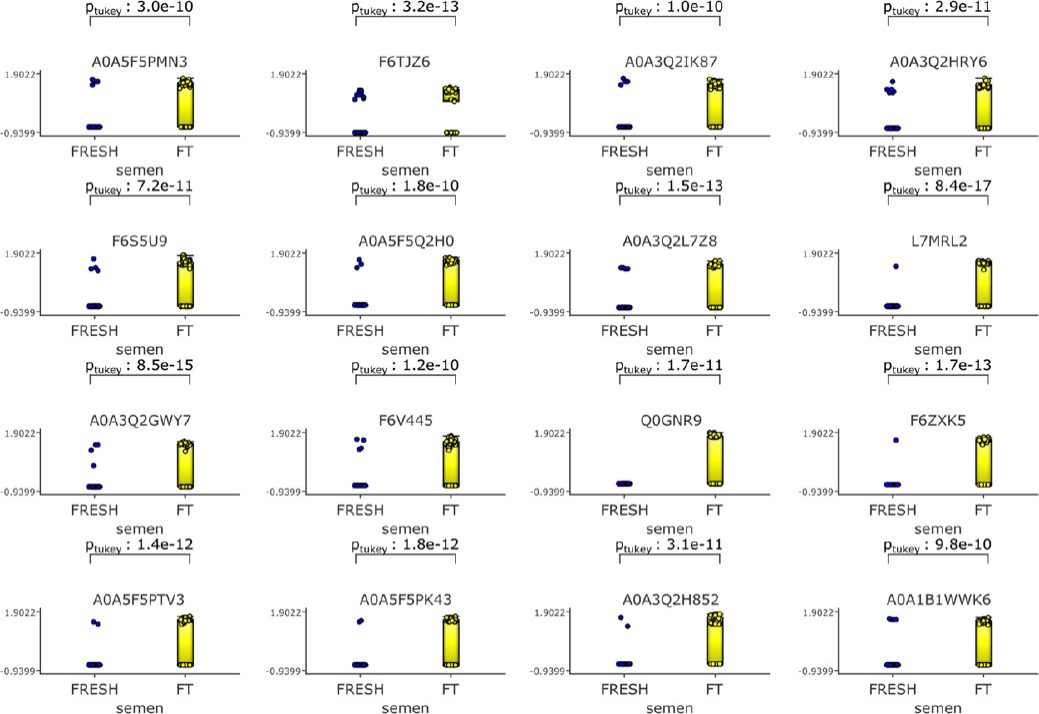
Direct comparison showing proteins which are more abundant in frozen thawed samples *p* = 8.00 × 10^–10^, *q* = 3.39 × 10^–9^ fold change >4. Qlucore Omics Explorer (Lund, Sweden https://qlucore.com) was used to compare differences in the relative amounts of proteins based on spectral counts between fresh and frozen thawed spermatozoa. Proteins in fresh spermatozoa are represented by blue circles, proteins in frozen thawed spermatozoa are represented by yellow circles.

Amounts of numerous proteins were increased as a result of cryopreservation (Figure 11). These were the *RNA polymerase III subunit A*, *polyribonucleotide nucleotidyltransferase 1*, *an uncharacterized transmembrane protein similar to a putative spermatogenesis-associated protein 31C1 in humans (66.7% similarity)*, *an uncharacterized protein, kinesin family member 27*, *class II major histocompatibility complex transactivator*,*G-protein coupled receptor 25*, *transmembrane protein 5 like protein*, *olfactory receptor*, *exosome component 2*, *HSPV163*, *cyclic nucleotide binding domain-containing protein*, *A kinase anchoring protein 7*, *TBC1 domain family member*, *checkpoint with forkhead and ring finger domain protein*, and *genome polyprotein*. The most statistically significant changes observed were in the amounts of *transmembrane protein 5 like protein* (*p* = 8.4 × 10^–17^) and the *olfactory receptor* (*p* = 8.5 × 10^–15^) (Figure 11).

### Cryopreservation Impairs Sperm Functionality and Causes Oxidative Stress

**Figure 12:**
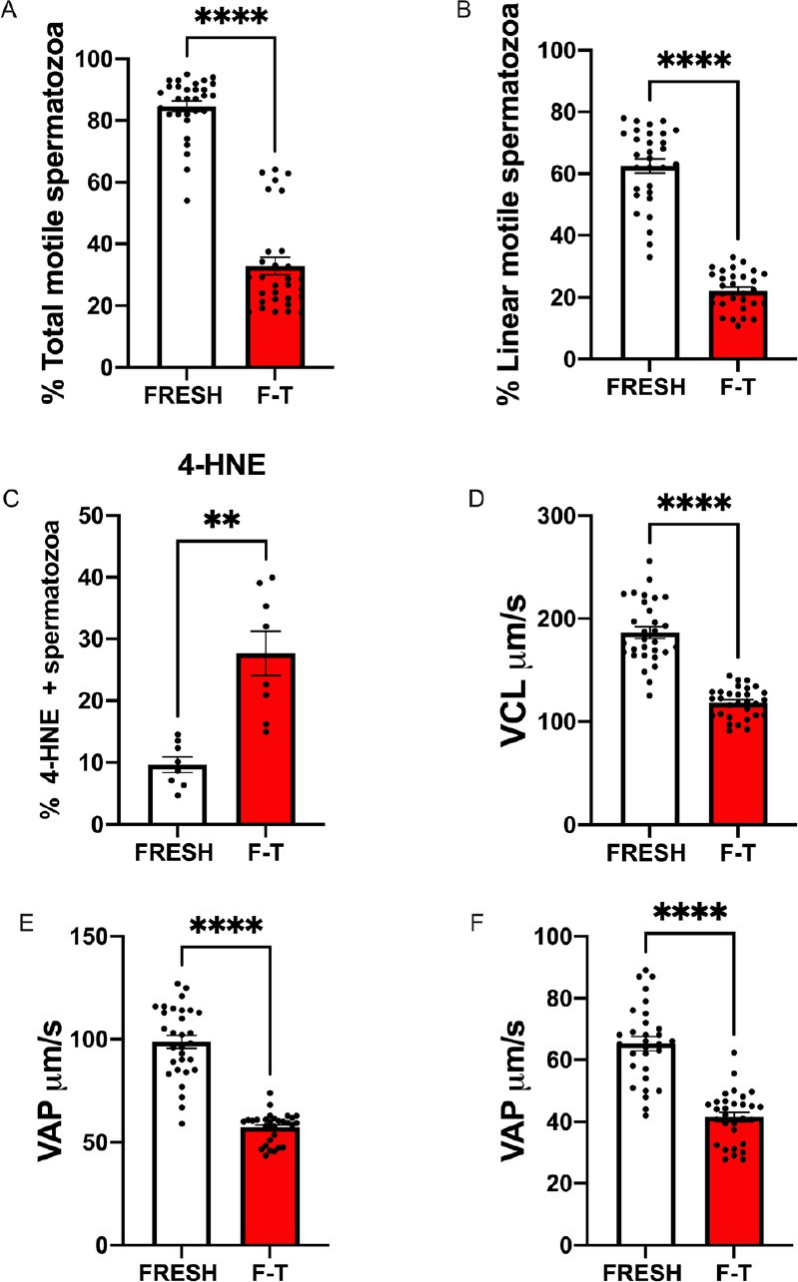
Impact of cryopreservation on sperm functionality. Stallions ejaculates were processed and analyzed as described in the Materials and Methods. Computer assisted sperm analysis (CASA) was used to assess sperm motility (% total and linear motile spermatozoa) and velocities, VCL (circular velocity) μm/s, VAP (average path velocity) μm/s, and straight line velocity (VSL) μm/s. Flow cytometry was used to determine spermatozoa experiencing lipid peroxidation (4-hydroxynonenal 4-HNE). Data are presented as means ± SEM. **** *P* < 0.0001, ** *P* < 0.01.

Freezing and thawing caused a major impact on sperm functionality. The percentage of total and linear motile spermatozoa dropped after cryopreservation from 84.6 ± 1.7 and 62.5 ± 2.2 in fresh samples to 32.8 ± 2.9 and 22.5 ± 1.1%, respectively, in frozen thawed samples (*P* < 0.0001) (Figure 12A,B). Significant reductions in sperm velocities were seen after cryopreservation; circular velocity (VCL) dropped from 186.5 ± 5.6 μm/s in fresh samples to 118.3 ± 14.6 μm/s in thawed samples (*P* < 0.0001) (Figure 12D). Also, straight line (VSL) and average path velocities (VAP) were equally affected by cryopreservation (Figure 12E,F). The percentage of spermatozoa showing detectable levels of the α,β-unsaturated hydroxyalkenal, 4-hydroxynonenal (4-HNE) that is produced by lipid peroxidation in cells, increased in cryopreserved samples, from 9.6 ± 1.2% in fresh spermatozoa to 27.6 ± 3.5% after thawing (*p* < 0.001) (Figure 12C).

## Discussion

Cryopreservation inflicts a major insult on stallion spermatozoa. Among other insults, spermatozoa suffer osmotic stress at freezing and then again during thawing. They also experience cryoprotectant toxicity. On average all these factors mean that 50% or more of the spermatozoa initially entering the process succumb to osmotic induced necrosis, mainly at thawing.^39,40^ The surviving population also experiences changes arising due to the effect of osmotic stress in the mitochondria causing increased production of mitochondrial reactive oxygen species (ROS), exhaustion of antioxidants, and finally oxidative stress leading to accelerated senescence of the spermatozoa and premature death.^41^ Major changes in the stallion sperm proteome as a consequence of cryopreservation were identified in line with previous reports in different species.^11,15,17,18,42^ The gene ontology (GO) terms enriched in proteins in higher amounts in fresh spermatozoa reflected the importance of metabolism and redox reactions in spermatozoa and the well documented importance of mitochondria in these cells. To the contrary, in frozen thawed spermatozoa only the KEGG pathway for RNA degradation was enriched. The specific proteins that experienced highly significant changes as a result of cryopreservation were also evaluated and thus can be discriminants for the major consequences of stress imposed by the procedure. Cryopreservation caused a marked and highly significant decrease in the amount of Superoxide dismutase (Cu–Zn) (SOD1), an enzyme which forms part of the first line of defense against oxidative stress in most organisms.^43−45^ Although it has previously been reported that this enzyme is affected by cryopreservation,^11,46^ this is the first time that SOD1 is identified as a discriminating variable using bioinformatic analysis, being one of the most highly significantly different proteins between fresh and frozen thawed semen. This finding strongly supports the theory that alterations in redox regulation and oxidative stress are a major factor involved in cryodamage as also seen in the present study, indicated by the increased level of 4-HNE in cryopreserved spermatozoa. Other proteins with oxidoreductase activity were also identified as discriminant variables. The aldo-keto-reductase family 1 member b (AKR1B1) was also identified using bioinformatic analysis as discriminant for frozen thawed semen. Among the activities of this protein, there are some with special importance in the context of sperm biotechnologies. These include catalysis of the NADPH-dependent reduction of carbonyls, detoxifying lipid-derived unsaturated carbonyls, such as crotonaldehyde, 4-hydroxynonenal, trans-2-hexenal, trans-2,4-hexadienal and glutathione-conjugates of carbonyls (GS-carbonyls) as well as catalysis of the reduction of diverse phospholipid aldehydes such as 1-palmitoyl-2-(5-oxovaleroyl)-*sn*-glycero-3-phosphoethanolamin (POVPC) and related phospholipid aldehydes that are generated from the oxidation of phosphatidylcholine and phosphatidylethanolamines.^47^ In this context the role of this enzyme in detoxifying 4-HNE, a compound that has been characterized as extremely toxic for the spermatozoa is particularly interesting,^48^ and cryopreservation has been demonstrated to cause significant increases in the content of 4-HNE in spermatozoa,^49^ as also were evident in the present study. Thus, results presented here establish a plausible molecular explanation to much of the molecular damage occurring after cryopreservation, that is the reduction of the amount of key antioxidant proteins. In view of these findings it seems logical that spermatozoa containing higher amounts of (AKR1B1) may survive better after cryopreservation. Lysozyme B (LYZLB) was also present in higher amounts in fresh samples. According to geneontology.org this protein is involved in three biological processes: defense response to Gram negative and positive bacterium and fusion of sperm to egg plasma membrane involved in single fertilization.^50^ This finding may constitute another factor that explains the reduced fertility observed with cryopreserved spermatozoa.^51^ A serine/threonine-protein phosphatase was also more abundant in fresh sperm; BLAST revealed that this equine protein has a 100% homology with the human serine/threonine-protein phosphatase PP1- alpha catalytic subunit, a protein which is essential for spermatogenesis and spermatozoa motility.^52^ Reduction in the amount of proteins after cryopreservation can be explained by the well reported effects of freezing and thawing in the spermatozoa, reduction of antioxidant proteins can be explained by exhaustion due to the oxidative stress occurring during the procedure,^53^ and reduction in the amounts of other proteins can be explained due to protein degradation due to the osmotic stress occurring during the freeze–thaw cycle.^13,15,18,54^

Cryopreservation caused a highly significant increase in the amounts of 12 proteins. Two proteins were the most highly significantly enriched in thawed samples. *Transmembrane protein like 5* was increased in thawed samples, probably due to the intense stress that the plasma membrane of the spermatozoa experiences during cryopreservation.^39,55^ The increase in the amount of the *olfactory receptor* in cryopreserved samples is also noteworthy. This is also a membrane protein, a member of the class A rhodopsin-like family of G proteins coupled receptors.^56,57^ The most likely reason for the increase in this membrane receptor in cryopreserved samples is the intense disruption of the sperm membrane caused by the procedure. Since spermatozoa are translationally and transcriptionally silent cells, increases in the amounts of proteins are difficult to explain. As previously indicated, the stress of cryopreservation may have facilitated the exclusion of some proteins from the membrane and/or proteins that have experienced post translational modifications such as phosphorylation. Changes in the secondary and/or tertiary structure of the proteins have been proposed as an explanation for the increase in amounts of specific proteins after cryopreservation;^18^ however, this issue warrants further research.

In conclusion, cryopreservation imposes a major change on the stallion sperm proteome. These changes provide a molecular explanation for the reduced fertility observed with the use of frozen thawed spermatozoa, and provide molecular targets to be explored, with the aim of improving current cryopreservation procedures. Cryopreservation impacts the major antioxidant protein SOD1. Particular attention should be paid to the energetic metabolism of the spermatozoa and the relation with redox regulation in these cells in order to improve current cryopreservation procedures.
